# Automated measurement of the disc-fovea angle based on DeepLabv3+

**DOI:** 10.3389/fneur.2022.949805

**Published:** 2022-07-27

**Authors:** Bo Zheng, Yifan Shen, Yuxin Luo, Xinwen Fang, Shaojun Zhu, Jie Zhang, Maonian Wu, Ling Jin, Weihua Yang, Chenghu Wang

**Affiliations:** ^1^School of Information Engineering, Huzhou University, Huzhou, China; ^2^Zhejiang Province Key Laboratory of Smart Management & Application of Modern Agricultural Resources, Huzhou University, Huzhou, China; ^3^The Laboratory of Artificial Intelligence and Bigdata in Ophthalmology, Affiliated Eye Hospital of Nanjing Medical University, Nanjing, China; ^4^Advanced Ophthalmology Laboratory (AOL), Robotrak Technologies, Nanjing, China

**Keywords:** disc-fovea angle, automatic measurement, deep learning, retinal images, artificial intelligence

## Abstract

**Purpose:**

To assess the value of automatic disc-fovea angle (DFA) measurement using the DeepLabv3+ segmentation model.

**Methods:**

A total of 682 normal fundus image datasets were collected from the Eye Hospital of Nanjing Medical University. The following parts of the images were labeled and subsequently reviewed by ophthalmologists: optic disc center, macular center, optic disc area, and virtual macular area. A total of 477 normal fundus images were used to train DeepLabv3+, U-Net, and PSPNet model, which were used to obtain the optic disc area and virtual macular area. Then, the coordinates of the optic disc center and macular center were obstained by using the minimum outer circle technique. Finally the DFA was calculated.

**Results:**

In this study, 205 normal fundus images were used to test the model. The experimental results showed that the errors in automatic DFA measurement using DeepLabv3+, U-Net, and PSPNet segmentation models were 0.76°, 1.4°, and 2.12°, respectively. The mean intersection over union (MIoU), mean pixel accuracy (MPA), average error in the center of the optic disc, and average error in the center of the virtual macula obstained by using DeepLabv3+ model was 94.77%, 97.32%, 10.94 pixels, and 13.44 pixels, respectively. The automatic DFA measurement using DeepLabv3+ got the less error than the errors that using the other segmentation models. Therefore, the DeepLabv3+ segmentation model was finally chosen to measure DFA automatically.

**Conclusions:**

The DeepLabv3+ segmentation model -based automatic segmentation techniques can produce accurate and rapid DFA measurements.

## Introduction

The optic disc and macula are normal physiological structures of the eye. The physiologic disc-fovea angle (DFA) is measured using the line connecting the geometric center of the optic disc to the macular center and the horizontal line passing through the geometric center of the optic disc. The DFA of patients with rotational strabismus is usually beyond the normal range; therefore, DFA measurement is an important tool for the diagnosis of rotational strabismus. The measurement of the rotational strabismus angle is divided into subjective and objective examinations; the former examination utilizes the Maddox method (1), Jackson crossed columnoscopy (2), and manual corneal margin marking (3), while the latter examination is measured using fundus images (4–8). Currently, DFA measurement based on fundus images are mainly measured manually and suffer from an average error of 2.0 (±1.8) (9). There were several disadvantages in manual measurement of DFA: poor reproducibility, low accuracy and time-consuming manual measurement (6, 10). Therefore, it is necessary to research automatic DFA measurement methods.

Artificial intelligence has applications in various fields. Since ophthalmology has many structured images, it has become one of the frontiers of artificial intelligence research (11, 12). AI has many applications in the field of classification and segmentation in ophthalmology (13–20). The automatic measurement of DFA is associated with optic disc segmentation (21–24), optic disc centration (25–27), and macular centration (28). Xiong et al. (29) investigated optic disc segmentation using the U-Net model. Bhatkalkar et al. (30) used a deep learning-driven heat map regression model to locate the optic disc center. Cao et al. (31) proposed a macular localization method based on morphological features and k-mean clustering. There are many related studies illustrate similar methods (32–35). At present, the DFA measurement method based on fundus images has been less studied. Simiera et al. proposed the Cyclocheck software, which measured DFA by locating the center of the macula and its tangent line to the optic disc (6). Piedrahita et al. (10) designed a DFA measurement software by using MATLAB language and the software needed manually locate the optic disc edge and macular center to calculate the DFA. The above methods were suitable for single-image measurement and required physician participation; its disadvantages include the requirement of step-by-step completion, poor repeatability, and low efficiency. To circumvent these weaknesses and challenges, this study designed a fully automated method for measuring the DFA using a deep learning model.

In this study, three segmentation models (DeepLabv3+, U-Net, and PSPNet) were trained to segment the optic disc area and virtual macular area based on 682 normal fundus images. The DFA was calculated by finding the macular and optic disc centers. Therefore, an automatic DFA measurement based on fundus images is realized.

## Materials and methods

### Data source

The color fundus image data from the Eye Hospital of Nanjing Medical University were obtained from non-mydriasis fundus cameras, and they collected from January to June 2021. A total of 682 images, each with a size of 2,584 × 1,985 and the angle of the color images were selected to be correct. To meet the inclusion criteria of this study, all of the eyes that were selected for the fundus images had no retinal disease, and this was confirmed by the ophthalmologist. There were no restrictions based on sex or age for this study. Additionally, all relevant personal information was removed to avoid the inappropriate disclosure of private information.

**Figure 1:**
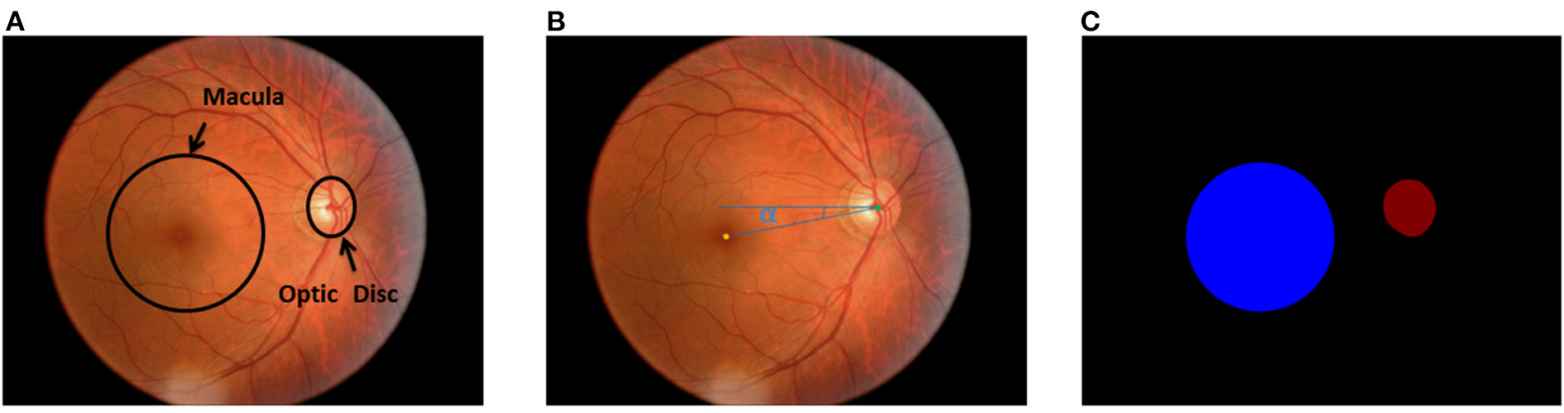
The normal fundus image and labeling map. **(A)** Normal fundus image, where the macular area is a virtual macular area labeled with the center of the macula as the circle and 400 pixels as the radius; **(B)** DFA; **(C)** Optic disc and virtual macular area labeling map[[Inline Image]].

The methodology of the study began with the labeling of the optic disc area, optic disc center and macular center. Then, the measurement of the true value of DFA using color fundus images was followed, which was performed by two professional ophthalmologists using the Adobe Photoshop software. Simultaneously, the virtual macular area label was obtained by using the macula center as the center of the circle, 400 pixels as the radius. Finally, all the labels were reviewed and confirmed by ophthalmologist. The normal fundus image is shown in Figure 1A, the DFA is shown in Figure 1B, and the fundus image and the optic disc and virtual macular area labeling map are shown in Figure 1C.

### Automatic measurement methods

**Figure 2:**
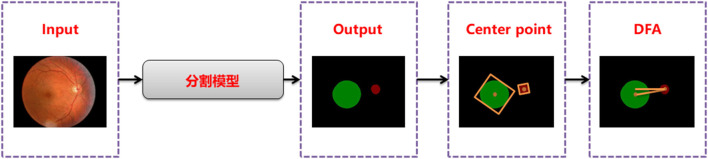
DFA automatic measurement flow chart.

Disc-fovea angle automatically measures the normal fundus image by inputting the image into the trained segmentation model to obtain the segmentation map of the optic disc and virtual macula area. Then, the minimum external matrix is used to obtain the central coordinates of the optic disc and macula. Combining the center coordinates with the inverse tangent function operation, the DFA is obtained by the radian operation. The workflow is shown in Figure 2.

### Segmentation model training

**Figure 3:**
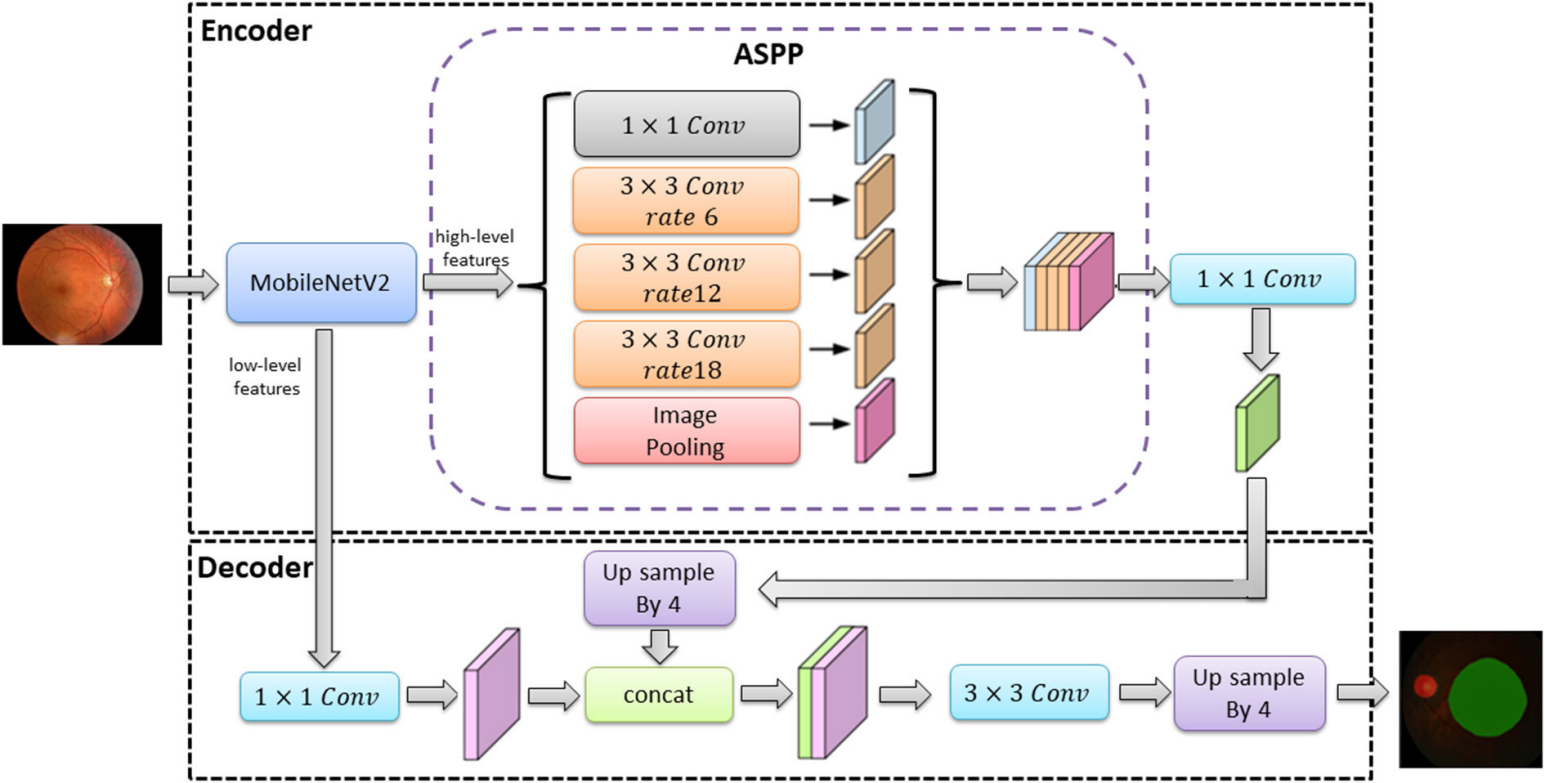
The segmentation model network structure of DeepLabv3+.

The classical deep learning segmentation models have U-Net, DeepLabv3+, and PSPNet. In this study, the DeepLabv3+ model was used to train the optic disc area and virtual macular area segmentation models using 682 normal fundus images. The images were divided into 477 images for training and 205 images for testing according to 7:3. The images used for training were divided into 429 images as training sets and 48 images as validation sets according to the ratio of 9:1.The model was trained with an image size of 512 × 512, a learning rate of 0.00005, and iterations time of summing up to a total of 100. The optimal optic disc and virtual macular area segmentation model was determined by identifying the model with the lowest loss in the validation set. The network structure of the DeepLabv3+ model is shown in Figure 3.

In Figure 3, the DeepLabv3+ model network structure is divided into an encoder and a decoder. First, the encoder extracts the image high-level and low-level features from the input image *via* the MobileNetV2 module. The high-level feature (the features representing the overall information of the image) are extracted by null convolution of different atrous rates in atrous spatial pyramid pooling (ASPP) before up sampling. The up sampling results are stitched with the low-level feature (the features of image boundary information) extracted from the MobileNetV2 module. The optic disc and virtual macular area prediction result map is obtained after convolution and up sampling.

**Figure 4:**
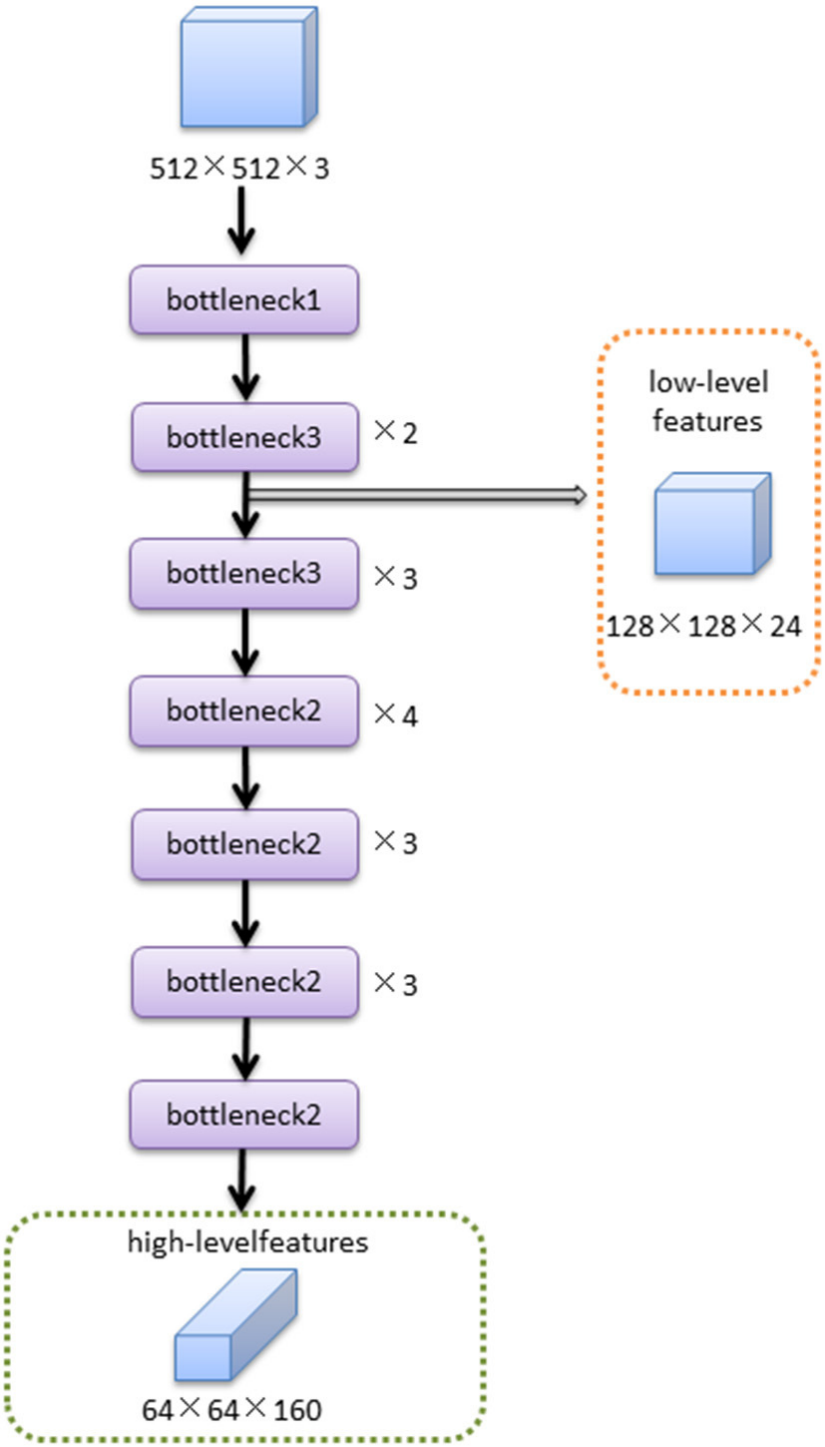
The feature extraction network of MobileNetV2.

**Figure 5:**
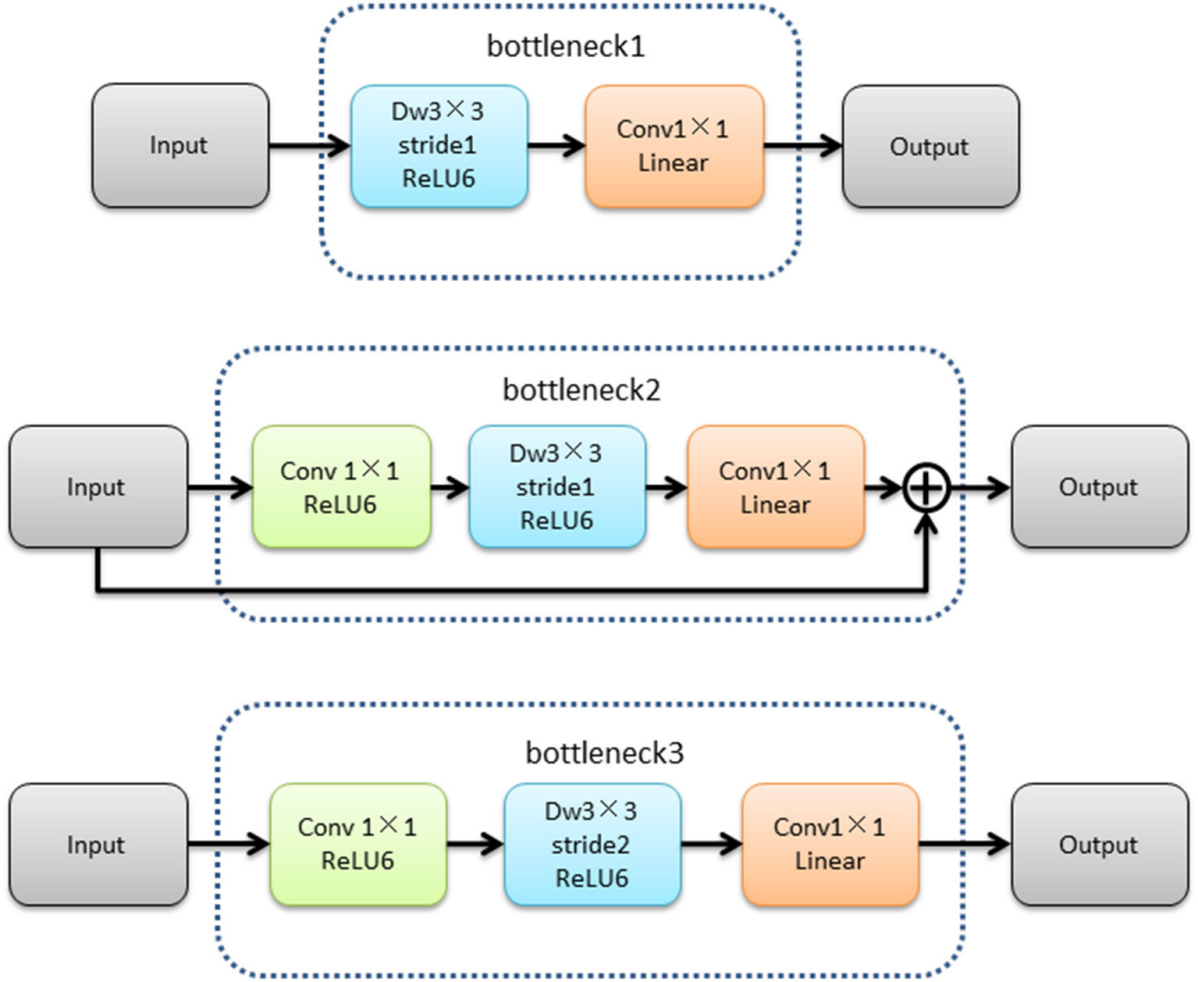
The structures of bottleneck1, bottleneck2, and bottleneck3.

The MobileNetV2 feature extraction network mainly extracted the features of input image. The low-level features were obtained after bottleneck1 and two bottleneck3 modules. The high-level features were obtained after 14 bottleneck modules followed low-level features. The structure of the MobileNetV2 feature extraction network was shown in Figure 4.The structures of bottleneck1, bottleneck2, and bottleneck3 in MobileNetV2 were show in Figure 5, they were all composed of convolution and deep convolution. When the input feature images and output feature images had same size in Bottleneck2 block, the output feature image was obtained by adding the input feature image and its convolution result, otherwise the output feature image was the convolution result.

The server hardware configuration used in this study was Intel (R) Xeon (R) silver 4214 CPU, the main frequency is 2.2 GHz, Tesla V100 graphics card, 32 GB video memory, and the operating system is Ubuntu 18 04. The deep learning framework is PyTorch and the programming language is Python.

### Calculation method

The DFA calculation formula is shown in Equation (1), where the optic disc center coordinates are (OX, OY) and the macular center coordinates are (MX, MY). If MY > OY, the DFA takes the opposite number.


(1) $$DFA=\frac{|\frac{OX-MX}{OY-MY}|\times 180}{\pi }.$$

The evaluation indexes for measuring the accuracy of the segmentation model in this study are the intersection over union (IoU), pixel accuracy (PA), MIoU, and MPA, as shown in Equations (2), (3), (4), and (5), respectively. In these equations, TP indicates the number of correctly predicted pixels in the optic disc and virtual macular areas, TN indicates the number of correctly predicted pixels in the background area, FP indicates the number of incorrectly predicted pixels in the optic disc and virtual macular areas, and FN indicates the number of incorrectly predicted pixels in the background area.


(2) $$\begin{array}{l} IoU=\frac{TP}{FN+FP+TP} \end{array}$$

(3) $$\begin{array}{l} PA=\frac{TP+TN}{FN+FP+TP+TN} \end{array}$$

(4) $$\begin{array}{l} MIoU=\frac{1}{k+1}\overset{k}{\underset{i=0}{\sum }}\frac{TP}{FN+FP+TP}\backslash n \end{array}$$

(5) $$\begin{array}{l} MPA=\frac{1}{k+1}\overset{k}{\underset{i=0}{\sum }}\frac{TP+TN}{FN+FP+TP+TN}\backslash n \end{array}$$

The optic disc center and macular centroid errors were calculated as follows: DO indicate the optic disc center error, and the formula is shown in Equation (6). DM indicates the macular center error, and the formula (in pixels) is shown in Equation (7). The following points are defined as: (OX1, OY1) as the true optic disc center, (MX1, MY1) as the true macular center, (OX2, OY2) as the segmented obtained optic disc center, and (MX2, MY2) as the segmented obtained macular center.


(6) $$\begin{array}{l} DO=\sqrt{{\left(OX2-OX1\right)}^{2}+{\left(OY2-OY1\right)}^{2}} \end{array}$$

(7) $$\begin{array}{l} DM=\sqrt{{\left(MX2-MX1\right)}^{2}+{\left(MY2-MY1\right)}^{2}.} \end{array}$$

## Results

**Figure 6:**
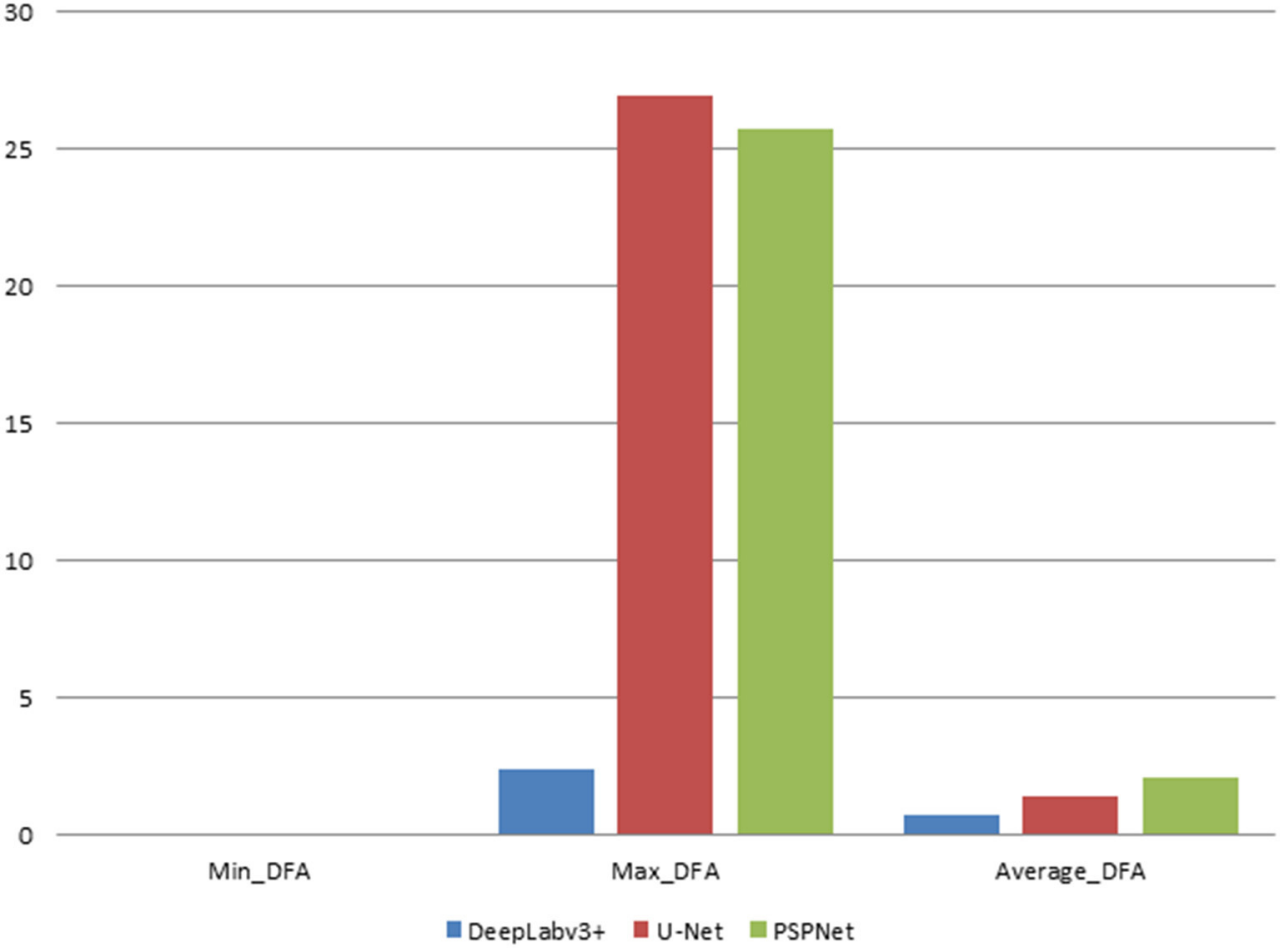
The statistical plots of DFA error results for the three segmentation models (deg).

In this study, the automatic DFA measurement method based on DeepLabv3+ was tested using 205 normal fundus images. The results were compared with the DFA angles obtained from the U-Net and PSPNet segmentation-based models. The comparison of the DFA errors obtained from the three models is shown in Table 1 and Figure 6. The automatic DFA measurement method based on the DeepLabv3+ segmentation model achieves the smallest average error of 0.76°, which is 0.66° and 1.36° lower than the errors obtained using the U-Net and PSPNet models, respectively.

**Table 1:** Three different segmentation models with error results of DFA (deg).

**Model**	**Min_DFA**	**Max_DFA**	**Average_DFA**
DeepLabv3+	0	2.42	0.76
U-Net	0.01	26.98	1.42
PSPNet	0.01	25.72	2.12

The disc-fovea angle (DFA) is in degrees (deg).

**Figure 7:**
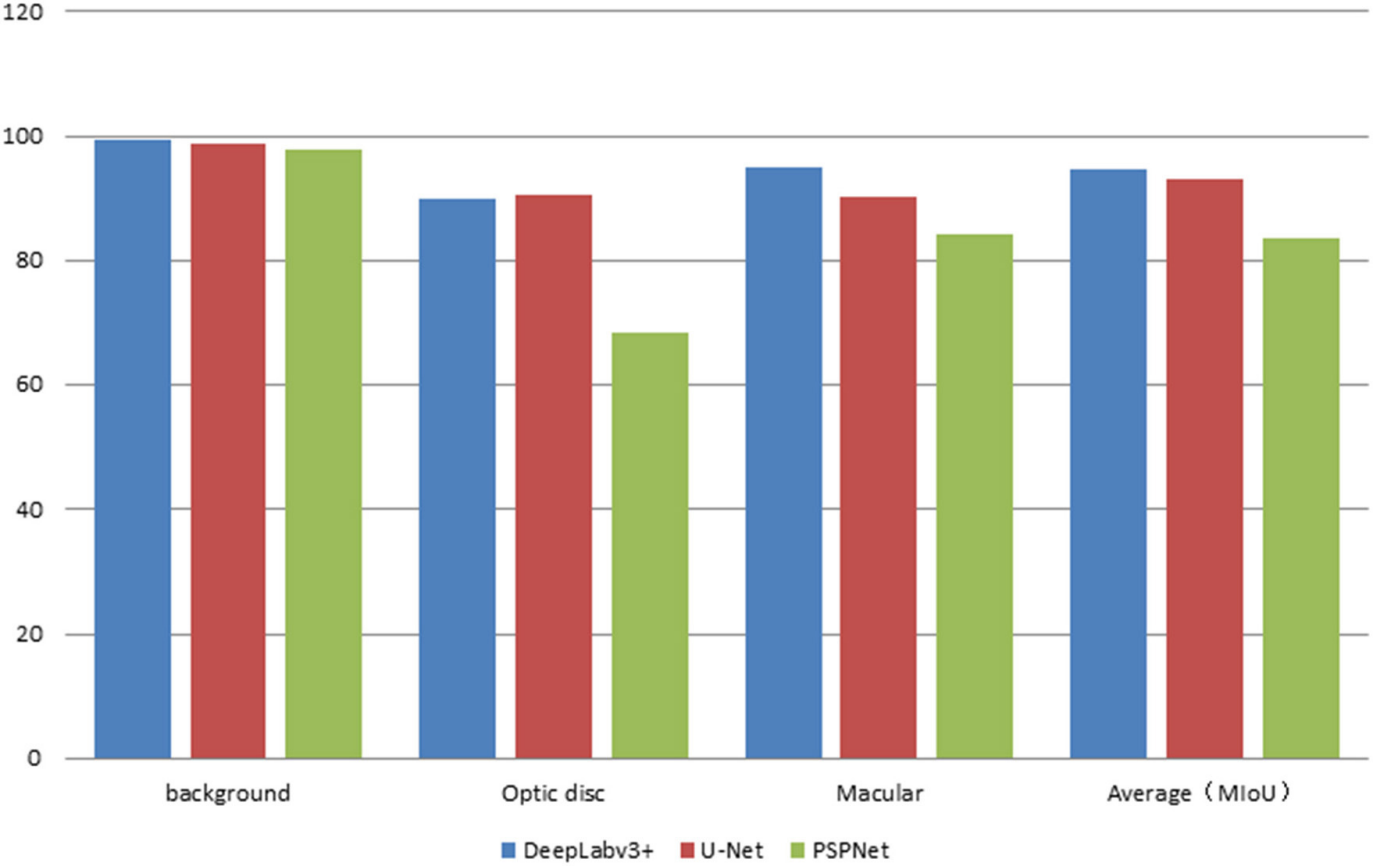
The statistics plots of IoU of the three segmentation models.

**Figure 8:**
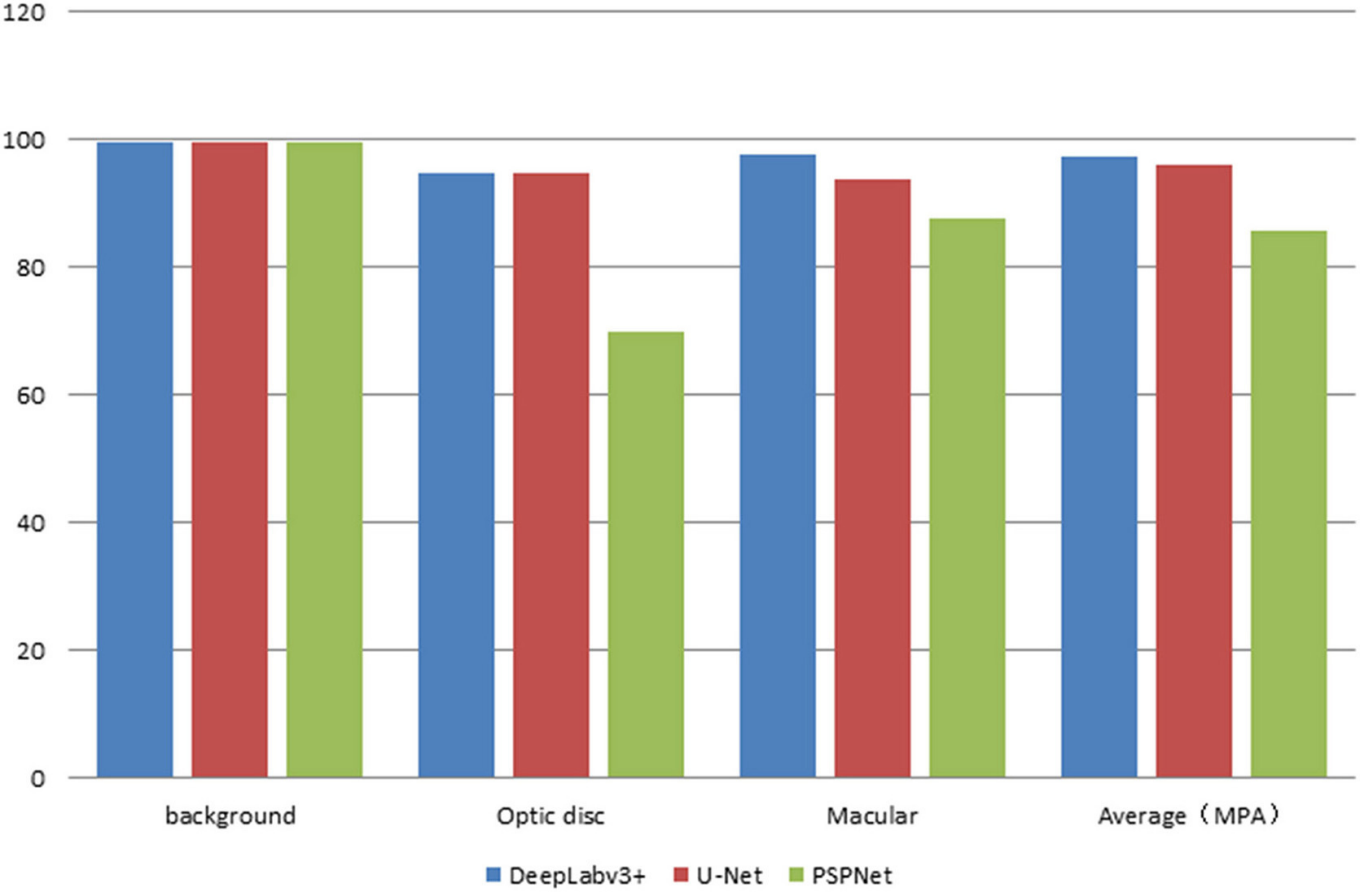
The statistics plots of PA of the three segmentation models.

**Figure 9:**
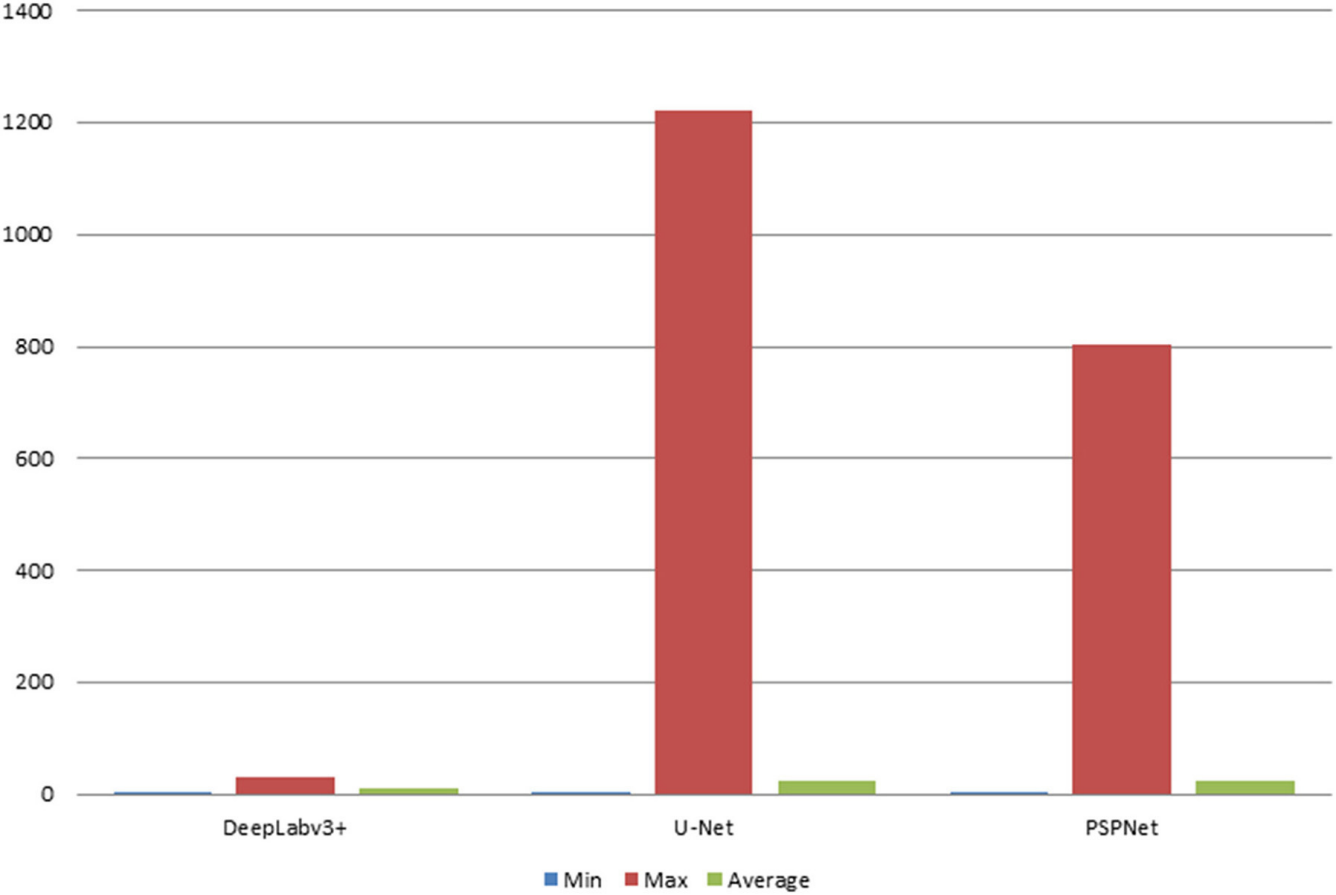
The statistical plots of DO of the three segmentation models (deg).

**Figure 10:**
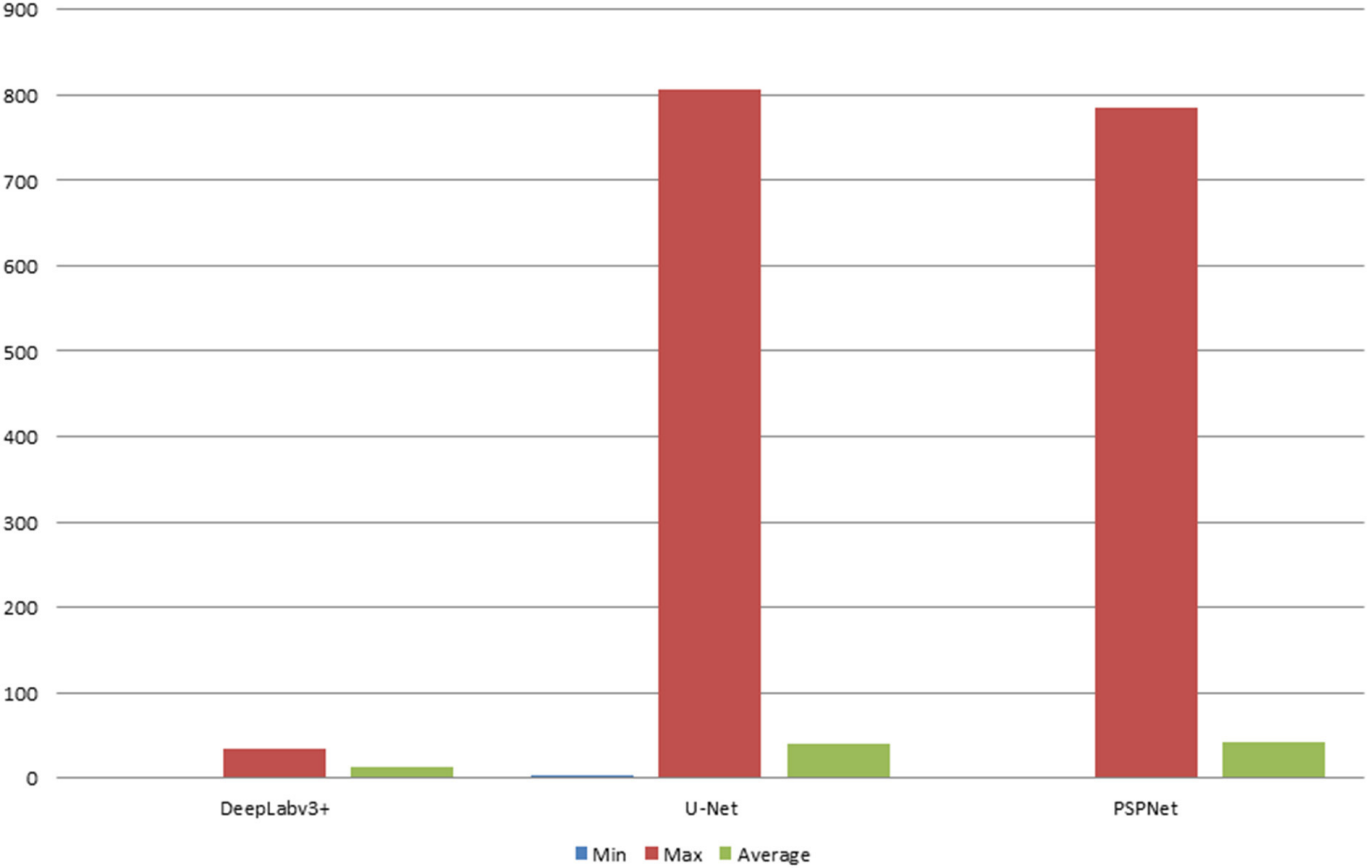
The statistical plots of DM of the three segmentation models (deg).

**Figure 11:**
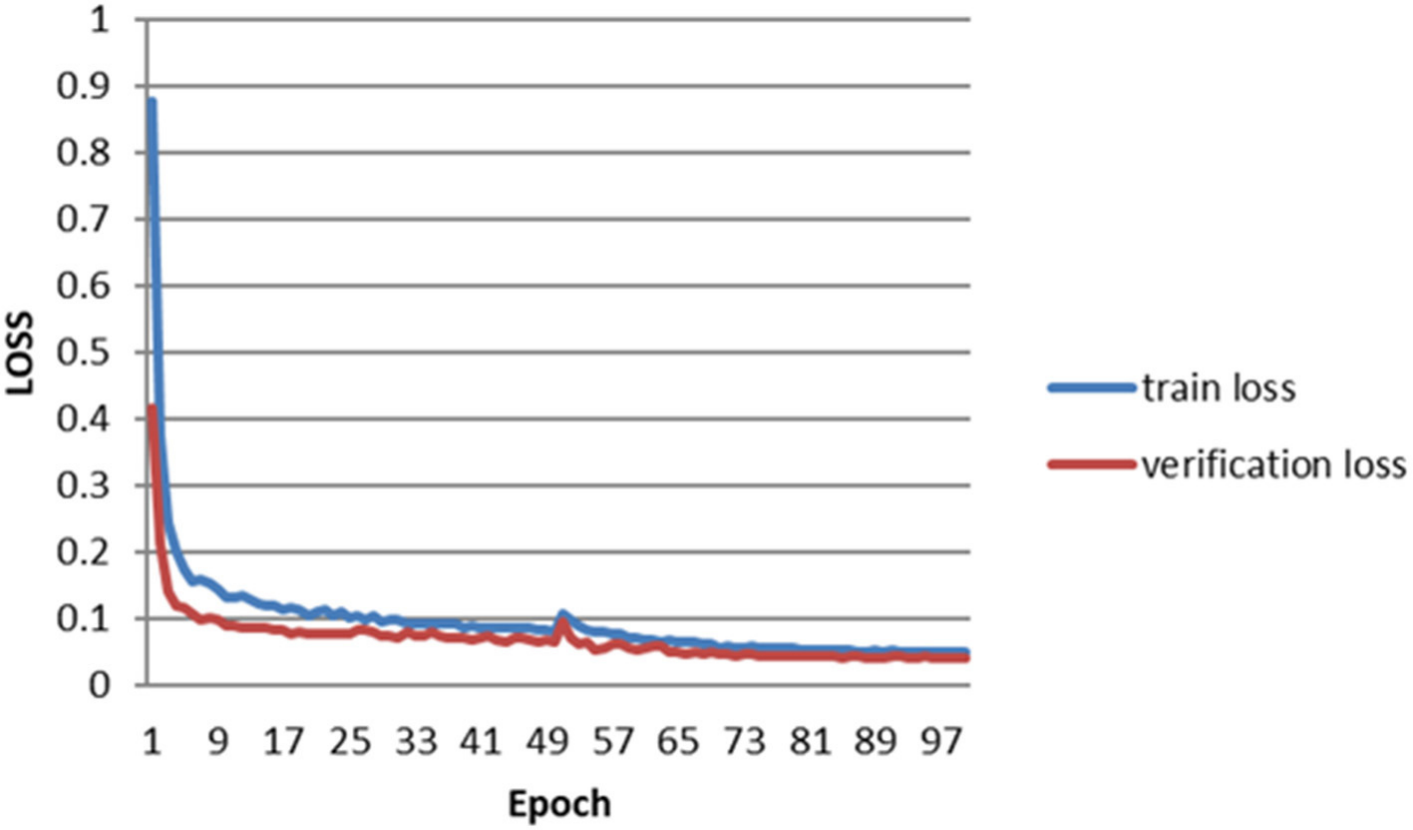
Loss curves of DeepLabv3+.

The error of the DFA is closely related to the accuracy of optic disc and virtual macular region segmentation. Therefore, the evaluation index of the three model segmentation results, the error values of optic disc center and macular center were also quantified and presented in Table 2, Figures 7, 8. The DeepLabv3+ segmentation model achieved the best results in MIoU and MPA. The result of MIoU was 1.6% higher than that for the U-Net segmentation model's and 11.26% higher than that for the PSPNet segmentation model's. As shown in Table 3, Figures 9, 10, the model was also optimal for the comparison between the error at the optic disc centroid and macular centroid, where the maximum error at the macular center was 34.54 pixels or ~0.239 mm (36). The loss curves for the DeepLabv3+ segmentation model are shown in Figure 11. The training and validation loss curves gradually stabilized with an increase in the epoch.

**Table 2:** Comparison of evaluation indicators of three segmentation models (%).

**Models**	**DeepLabv3**+		**U-Net**		**PSPNet**	
**Evaluation indicators**	**IoU**	**PA**	**IoU**	**PA**	**IoU**	**PA**
Background	99.34	99.66	98.79	99.51	97.89	99.53
Optic disc	89.93	94.74	90.61	94.67	68.48	69.86
Macular	95.05	97.56	90.1	93.76	84.15	87.68
Average (MIoU/MPA)	94.77	97.32	93.17	95.98	83.51	85.69

IOU, intersection over union; PA, pixel accuracy; MIoU, mean intersection over union; MPA, mean pixel accuracy.

**Table 3:** Comparison of optic disc centroid and macular centroid errors for the three models (pixels).

**Models**	**DeepLabv3**+		**U-Net**		**PSPNet**	
**Evaluation indicators**	**DO**	**DM**	**DO**	**DM**	**DO**	**DM**
Min	0.36	1.36	0.73	3.38	0.41	2.01
Max	30.34	34.54	1221	805.62	803.08	785.69
Average	10.94	13.44	25.8	39.24	25.08	41.29

DO, optic disc center error; DM, macular center error.

This study segmented the optic disc and macula of the fundus image using three segmentation models. The DFA was calculated by finding the centers of both separately for the segmentation image. Among the three segmentation methods, the average error of DFA obtained by the automatic DFA measurement method based on DeepLabv3+ was 0.76°, which is 1.24° smaller than the average error of 2.0 (±1.8) ° measured manually.

## Discussion

Currently, DFA measurement method is still a time-consuming manual measurement method performed by ophthalmologist; it is inefficient and poorly reproducible. With the developments of artificial intelligence in medicine, the levels of automation and intelligence have increased, and some semi-automatic methods have been established to measure DFA automatically by manually positioning the optic disc and macular center. However, this method still requires each step to be performed by an ophthalmologist, and it is still a manual measurement technique. For this reason, fully automated DFA measurement methods have high research value.

The DeepLabv3+ - based DFA auto-measurement method can obtain the best results when compared to the U-Net and PSPNet models. The DeepLabv3+ model fuses multiscale information in the form of an encoder and decoder. The fundus image is input to the MobileNetV2 and obtains low-level feature layers (boundary information) of size 64 × 64 and high-level feature layers (semantic information) of size 128 × 128. The features of the two feature layers are extracted and fused to improve the boundary segmentation accuracy. Therefore, the model can extract features more adequately, segment boundaries with higher accuracy, and ultimately obtain smaller DFA measurement errors.

Existing DFA measurement methods are mainly manual or semi-automatic (37). Simiera et al. DFA was measured by Cyclocheck software. The DFA was calculated by manually importing a single fundus image and drawing two separate tangents to the top and bottom of the optic disc based on the localized macular center (6). Piedrahita et al. The DFA was calculated by manually acquiring the optic disc edge and macular center. The mean absolute difference between the repeated measurements was 1.64° (10).These semi-automatic methods still need to be operated by ophthalmologist and they are poor repeatability, low accuracy, and time-consuming. The automatic DFA measurement utilized in this study can directly obtain DFA values after inputting fundus images, thus making it more efficient and accurate.

The MIoU and MPA values for segmenting the optical disc and virtual macula using the DeepLabv3+ segmentation model were 95.7 and 97.32%, respectively. The automatic DFA measurement based on this model results in an error of 0.76°, which can be attributed to insufficient training data. It is important to consider that there are only a few ophthalmology-related public databases and make deep learning models difficult to train (38). Moreover, this study only used 682 normal fundus images provided by the partner hospital, which contributed to the less accurate segmentation results. It will increase the training data and improve the segmentation model to improve the accuracy of DFA measurement in the future.

All the images used in this study were obtained from normal eyes. Considering that it is difficult to label fundus images in diseased eyes, it will also be difficult to locate the optic disc and macular centers accurately. Therefore, this study did not include them in the initial automatic DFA measurement.

## Conclusion

There were three segmentation models were used to obtain optic disc-virtual macular segmentation results, and DFA values were further obtained by calculation. Among the three segmentation methods, DFA based on DeepLabv3+ had the least average error, which was 0.76°. The automatic measurement of DFA based on DeepLabv3+ can obtain more objective results, assist ophthalmologists to quickly measure DAF value, improve measurement efficiency, and reduce the burden on ophthalmologists. This study mainly studied the automatic DFA measurement of normal fundus. In the future, the relevant data of diseased fundus will be collected and the automatic DFA measurement of diseased fundus will be studied.

## Data availability statement

The datasets presented in this article are not readily available as requested of the partner hospital. Requests to access the datasets should be directed at: WY, benben0606@139.com. And I did not detect any particular expressions.

## Author contributions

BZ and YS wrote the manuscript. BZ planed experiments and the manuscript. SZ guided the experiments. MW, WY, and CW reviewed the manuscript. YS and XF trained the model. YL, JZ, and LJ collected and labeled the data. All authors issued final approval for the version to be submitted.

## Funding

The study supported by the National Natural Science Foundation of China (No. 61906066), Natural Science Foundation of Zhejiang Province (No. LQ18F020002), Hospital Management Innovation Research Key Project of Jiangsu Provincial Hospital Association (JSYGY-2-2021-467), the Ningbo Medical Science and Technique Program (2021Y71), and the Medical Big Data Clinical Research Project of Nanjing Medical University, Postgraduate Research and Innovation Project of Huzhou University (No. 2022KYCX35).

## Conflict of interest

The authors declare that the research was conducted in the absence of any commercial or financial relationships that could be construed as a potential conflict of interest.

## Publisher's note

All claims expressed in this article are solely those of the authors and do not necessarily represent those of their affiliated organizations, or those of the publisher, the editors and the reviewers. Any product that may be evaluated in this article, or claim that may be made by its manufacturer, is not guaranteed or endorsed by the publisher.
